# Perspectives on Private Equity Investment in Assisted Living

**DOI:** 10.1093/geroni/igaf122.1408

**Published:** 2025-12-31

**Authors:** Paula Carder, Jacklyn Kohon, Sarah Dys, John Bowblis, Yashaswini Singh, Sean Huang, Momotazur Rahman, Kali Thomas

**Affiliations:** Portland State University, Portland, Oregon, United States; Portland State University, Portland, Oregon, United States; Portland State University, Portland, Oregon, United States; Miami University, Oxford, Ohio, United States; Brown University School of Public Health, Providence, Rhode Island, United States; Georgetown University, Washington, District of Columbia, United States; Brown University, Providence, Rhode Island, United States; Johns Hopkins University, Baltimore, Maryland, United States

## Abstract

This qualitative study on the role of private equity (PE) in assisted living mergers, acquisitions, and development, included open-ended interviews with 26 senior housing professionals, 10 state agents, and 7 ombuds offices. Participants, identified through a combination of standard qualitative approaches, including referrals from study team networks, snowball sampling, grey literature review, and state agency, senior housing and investment firm websites. The senior housing professionals included 8 owner/operators, 5 PE fund managers, 5 lenders, 2 real estate attorneys, 2 senior housing consultants, 2 senior housing professional organizations, a real estate developer, and an insurance broker. Interviews averaged 60 minutes, were conducted online, audio-recorded and transcribed. We used “practical” thematic analysis (Saunders, 2022): first, independently reading transcripts, developing and applying descriptive codes, and writing summary memos; and second, as a team, discussing data saturation, reviewing coding, and co-constructing themes. All participants were asked to define PE, and describe challenges and benefits of PE involvement. We present overarching themes that describe areas of alignment and divergence across participant types. Themes that describe areas of alignment: Local operators provide higher quality care compared to national firms, AL combines real estate and healthcare, AL faces significant workforce challenges and capital investments result in improvements. Themes that describe divergence: Changes following a PE-funded acquisition are positive or negative, PE investment impacts affordability, the demand for PE-backed senior housing, and what attracts investors. This paper provides a unique perspective on how AL communities are developed and operated, and on mergers and acquisitions.

